# Analysis of the relationship between the gut microbiome and dementia: a cross-sectional study conducted in Japan

**DOI:** 10.1038/s41598-018-38218-7

**Published:** 2019-01-30

**Authors:** Naoki Saji, Shumpei Niida, Kenta Murotani, Takayoshi Hisada, Tsuyoshi Tsuduki, Taiki Sugimoto, Ai Kimura, Kenji Toba, Takashi Sakurai

**Affiliations:** 1 Center for Comprehensive Care and Research on Memory Disorders, National Center for Geriatrics and Gerontology, Aichi, Japan; 2 Medical Genome Center, National Center for Geriatrics and Gerontology, Aichi, Japan; 3 Biostatistics Center, Graduate School of Medicine, Kurume University, Fukuoka, Japan; 4 TechnoSuruga Laboratory Co., Ltd, Shizuoka, Japan; 5 Laboratory of Food and Biomolecular Science, Department of Bioscience and Biotechnology for Future Bioindustries, Graduate School of Agricultural Science, Tohoku University, Miyagi, Japan; 6 Department of Cognition and Behavioural Science, Nagoya University Graduate School of Medicine, Aichi, Japan

## Abstract

Dysregulation of the gut microbiome is associated with several life-threatening conditions and thus might represent a useful target for the prevention of dementia. However, the relationship between the gut microbial population and dementia has not yet been fully clarified. We recruited outpatients visiting our memory clinic to participate in this study. Information on patient demographics, risk factors, and activities of daily living was collected, and cognitive function was assessed using neuropsychological tests and brain magnetic resonance imaging scans. Faecal samples were obtained, and the gut microbiome was assessed by terminal restriction fragment length polymorphism (T-RFLP) analysis, one of the most well-established and reliable 16S ribosomal RNA-based methods for classifying gut microbiota. Patients were divided into two groups, demented and non-demented. Multivariable logistic regression models were used to identify the variables independently associated with dementia. The T-RFLP analysis revealed differences in the composition of the gut microbiome: the number of *Bacteroides* (enterotype I) was lower and the number of ‘other’ bacteria (enterotype III) was higher in demented than non-demented patients. Multivariable analyses showed that the populations of enterotype I and enterotype III bacteria were strongly associated with dementia, independent of the traditional dementia biomarkers. Further studies of the metabolites of gut microbes are needed to determine the mechanism underlying this association.

## Introduction

Globally, 47 million people were living with dementia in 2015, and this number is projected to triple by 2050^1^. Dementia is an important healthcare problem in Japan, where by the mid-2010s, 4 million people, representing 15% of the over-65 population, had received a diagnosis of dementia^2^. Therefore, a comprehensive strategy for dementia research has been introduced to improve future healthcare in Japan^3^. Assessment of the risk of dementia from various viewpoints is useful because a multifactorial approach is important to determine the essential mechanisms underlying the disease.

Recently, differences in the gut microbiome have been found to be associated with several life-threating conditions, such as obesity, cardiovascular diseases and inflammatory diseases^4–6^. The gut microbiome can be defined as all of the species within the ecosystem and it is the largest reservoir of microbes in the human body, consisting of ~10^14^ cells^7^. Furthermore, recent research has identified a novel association between the gut microbiome and dementia^7,8^, suggesting that the gut microbiome may modulate host brain function *via* a microbiome–gut–brain axis^9^. The existence of such an axis has been hypothesised because changes in the gut microbiome have been shown to be part of the mechanism linking high levels of fat consumption and other unbalanced diets with impaired cognition^9^. More specifically, disruption of the neuro-inflammatory system may be caused by gut microbes^10^, which could lead to the deposition of amyloid β in the brain^11,12^. This may be a key component of the pathogenesis and progression of dementia, and particularly of Alzheimer’s disease. Nevertheless, the mechanism is still unclear and the composition of the gut microbiome differs among individuals according to their race and diet. Previous studies have revealed an association between the gut microbiome and cardiovascular disease in Japanese patients^5,6^; however, it remains to be established whether the composition of the gut microbiome is associated with dementia in the Japanese population.

In the present study, we aimed to investigate the relationship between the composition of the gut microbiome and dementia in Japanese patients, using a comprehensive assessment of cognitive function. We hypothesised that there would be differences in the composition of the gut microbiome between demented and non-demented patients.

## Results

### Patient characteristics

Informed consent was obtained from 181 patients who visited the memory clinic at the National Center for Geriatrics and Gerontology (NCGG) during the study period. Of these, 53 were excluded owing to missing data or faecal samples (n = 27), an incomplete neuropsychological assessment (n = 22) or a decline in the condition of the patient (n = 4). Therefore, we analysed 128 eligible patients (female: 59%, mean age: 74.2 ± 8.7 years, mean Mini Mental State Examination [MMSE] score 24). The patients were stratified by their level of cognitive function: 94 were classified as non-demented and 34 as demented. There were 14 participants in the demented group that had a CDR of 0.5 and a MMSE of less than 20.

### Demented vs. non-demented patients

Compared with non-demented patients, demented patients were more likely to be female and to show fewer activities of daily living (ADL) and lower cognitive function (Table 1). Dementia patients scored lower on ADL, the Mini-Nutritional Assessment–Short Form (MNA-SF), MMSE, Raven’s Coloured Progressive Matrices, Frontal Assessment Battery and Logical Memory subtests I and II of the Wechsler Memory Scale-Revised; and scored higher on the Clinical Dementia Rating (CDR) scale, Dementia Behaviour Disturbance Scale, Zarit Caregiver Burden Interview and Alzheimer’s Disease Assessment Scale–cognitive subscale. Brain abnormalities, such as silent lacunar infarcts (SLIs) and cerebral microbleeds (CMBs), and high voxel-based specific regional analysis system for Alzheimer’s disease (VSRAD) scores, were frequent on magnetic resonance imaging (MRI) scans of patients with dementia. There were no significant differences in the laboratory findings, pulse wave velocity or ankle brachial index between the two groups, other than in estimated glomerular filtration rate (eGFR; demented 61.7 *vs*. non-demented 70.7 mL/min/1.73 m^2^, *P* = 0.028, Table S1). Demented patients were more likely to be taking anti-dementia drugs and anti-hyperglycaemic drugs than non-demented patients (25.7% *vs*. 6.5%, *P* = 0.005; 23.5% *vs*. 8.6%, *P* = 0.035, respectively). However, there were no significant differences in the numbers of patients taking other medications, such as anti-hypertensive drugs, statins, anti-thrombotic drugs, proton pump inhibitors/H2 blockers or aperients, between the two groups (Table S1).

**Table 1:** Patient characteristics.

	Total (*n* = 128)	Demented (*n* = 34)	Non-Demented (*n* = 94)	*P*
***Demographics***				
Age, years	76, 69–81	77, 74–82	76, 68–80	0.093
Female sex, n (%)	75 (58.6)	29 (85.3)	46 (48.9)	<0.001
Education, years	12, 9–12.8	12, 9–12	12, 9–13	0.456
Body mass index, kg/m^2^	22.6, 20.7–24.6	22.5, 20.3–25.0	22.7, 21.0–24.4	0.765
***Risk factors***				
Hypertension, n (%)	80 (62.5)	25 (73.5)	55 (58.5)	0.150
Diabetes mellitus, n (%)	20 (15.6)	8 (23.5)	12 (12.8)	0.169
Dyslipidaemia, n (%)	60 (46.9)	19 (55.9)	41 (43.6)	0.236
CKD, n (%)	41 (32.0)	14 (41.2)	27 (28.7)	0.203
Ischaemic heart disease, n (%)	13 (10.2)	5 (14.7)	8 (8.5)	0.329
History of stroke, n (%)	11(8.0)	4 (11.8)	7 (7.5)	0.481
Smoking habits, n (%)	32 (25.0)	3 (8.8)	29 (30.9)	0.011
Alcohol consumption, n (%)	49 (38.3)	10 (29.4)	39 (41.5)	0.303
ApoE ε4 carrier, n (%)	39 (30.5)	19 (55.9)	20 (21.3)	<0.001
***Comprehensive geriatric assessment***				
Barthel index	100, 100–100	100, 95–100	100, 100–100	0.009
IADL impairment, n (%)	59 (46.1)	26 (76.5)	33 (35.1)	<0.001
DBDS	9, 4–14	12.5, 7–18.3	7, 3–14	0.002
GDS	3, 1–5	3, 1–5	3, 1–5	0.730
Vitality index	10, 10–10	9, 8–10	10, 9–10	0.005
ZBI	11, 4–22	20.5, 13.5–28.3	7, 3–17.3	<0.001
MNA-SF	12, 11–13	12, 11–13	13, 11–13	0.049
***Cognitive function***				
MMSE	24, 20–28	18, 15–19	27, 23–29	<0.001
CDR-GB				<0.001
0, n (%)	23 (18)	0	23 (24.5)	
0.5, n (%)	85 (66.4)	14 (41.1)	71 (75.5)	
1, n (%)	18 (14.0)	18 (52.9)	0	
2, n (%)	1 (0.8)	1 (2.9)	0	
3, n (%)	1 (0.8)	1 (2.9)	0	
CDR-SB	2.0, 0.5–3.5	4.5, 3.4–5.6	1.0, 0.5–2.5	<0.001
ADAS-cog	9.3, 5.7–14.7	15.7, 12.9–20.2	7.5, 5–11.7	<0.001
RCPM	28, 23.3–31.8	25, 19–28	29, 24–32.5	<0.001
FAB	11, 9–13	9, 7–10	12, 10–14	<0.001
LM-WMSR I	8, 4–15	3, 1–5	10, 6–18	<0.001
LM-WMSR II	3, 0–8	0, 0–0	4.5, 1–10	<0.001
***Brain MRI findings***				
SLI, n (%)	14 (10.9)	9 (26.5)	5 (5.3)	0.002
WMH, n (%)	34 (26.6)	9 (26.5)	25 (26.6)	1.000
CMBs, n (%)	28 (21.9)	13 (38.2)	15 (16.0)	0.014
CSS, n (%)	8 (6.3)	4 (11.8)	4 (4.3)	0.207
VSRAD	1.02, 0.65–1.94	2.05, 1.16–2.32	0.85, 0.57–1.31	<0.001
***Blood flow reduction in SPECT images***				
Posterior cingulate gyrus and/or precuneus, n (%)	86 (71.1)	26 (81.3)	60 (67.4)	0.175
***Gut microbiota***				
Enterotype				0.001
Enterotype I	47 (36.7)	5 (14.7)	42 (44.7)	
Enterotype II	5 (3.9)	0	5 (5.3)	
Enterotype III	76 (59.4)	29 (85.3)	47 (50.0)	
F/B ratio	1.5, 1.0–2.4	2.1, 1.3–3.0	1.4, 0.8–2.3	0.013

Abbreviations: CKD, chronic kidney disease; MMSE, Mini Mental State Examination; CDR-GB, Clinical Dementia Rating Global Score; CDR-SB, Clinical Dementia Rating–sum of boxes; ADAS-cog, Alzheimer’s Disease Assessment Scale-Cognitive Subscale; RCPM, Raven’s Coloured Progressive Matrices; FAB, Frontal Assessment Battery; LM-WMSR, Logical Memory subtests I and II of the Wechsler Memory Scale-Revised; IADL, instrumental activities of daily living; DBDS, Dementia Behaviour Disturbance Scale; GDS, Geriatric Depression Scale; ZBI, Zarit Caregiver Burden Interview; MNA-SF, Mini-Nutritional Assessment-Short Form; SLI, silent lacunar infarct; WMH, white matter hyperintensity; CMBs, cerebral microbleeds; CSS, cortical superficial siderosis; VSRAD, voxel-based specific regional analysis system for Alzheimer’s disease; SPECT, single photon emission computed tomography; F/B ratio, Firmicutes/Bacteroidetes ratio. Enterotype I: Bacteroides >30%, Enterotype II: Prevotella >15%, enterotype III: others. The number of assessed patients: SPECT (*n* = 121), ADAS (*n* = 113), RCPM (*n* = 120), FAB (*n* = 122).

### Composition of the gut microbiome

**Figure 1:**
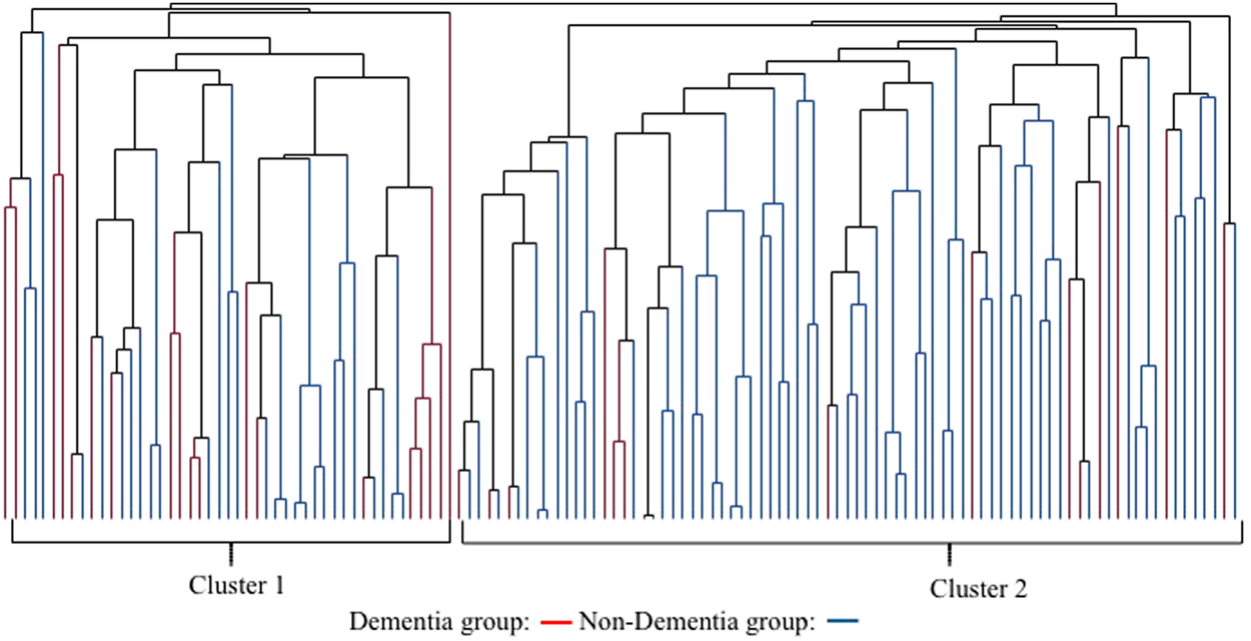
Dendrogram of the gut microbiome. A comparison of the gut microbiome between demented and non-demented patients demonstrates two major clusters of microbial taxa.

**Figure 2:**
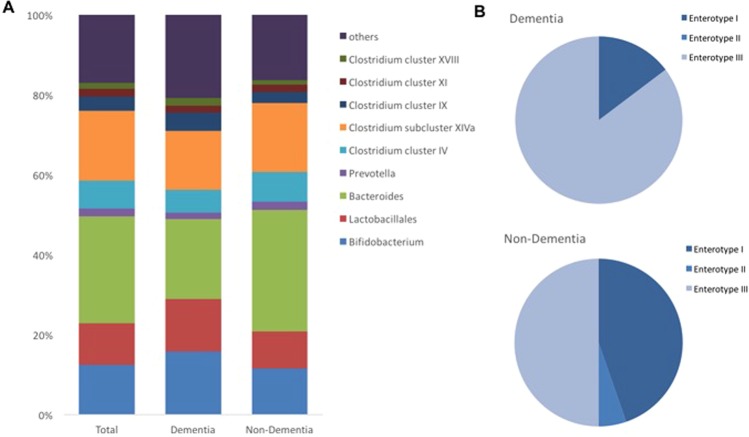
(**A**) Distribution of the gut microbiota. (**B**) Frequency of each enterotype in the dementia and non-dementia groups. Enterotype I (*Bacteroides* >30%) was enriched in the non-dementia group.

**Figure 3:**
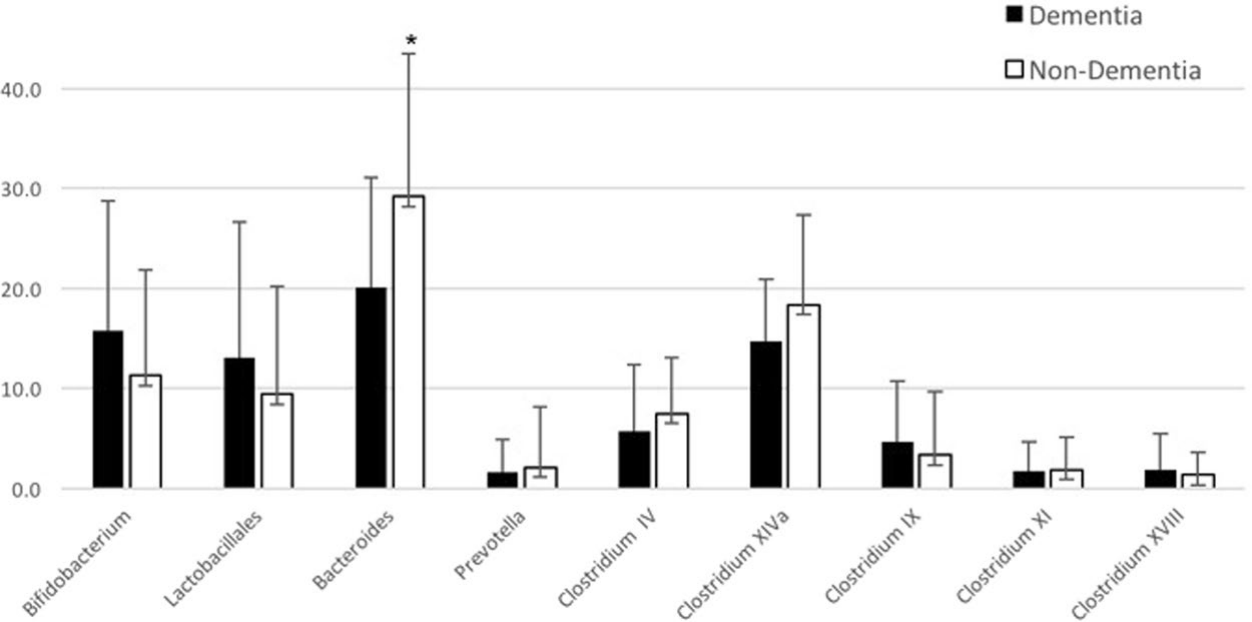
Comparison of the gut microbiota between the dementia and non-dementia groups. The percentages of each taxon of gut microbe was compared. The percentage of *Bacteroides* was significantly lower in the dementia than the non-dementia group.

The dendrogram comparing the gut microbiome between demented and non-demented patients showed two major clusters (Fig. 1). Cluster 1 contained more patients with dementia than cluster 2 (40.4% *vs*. 18.5%, *P* = 0.012, Fisher’s exact test). Terminal restriction fragment length polymorphism (T-RFLP) comparisons of the gut microbiome composition showed that this differed between demented and non-demented patients (Fig. 2A). More specifically, demented patients had fewer microbes of enterotype I and more of enterotype III than non-demented patients, suggesting a lower prevalence of *Bacteroides* and a higher prevalence of ‘other’ bacteria (*P* < 0.001, Fig. 2B). We also found that Lactobacillales and *Bifidobacterium* were slightly more frequent (Fig. 3). Furthermore, the Firmicutes/Bacteroidetes (F/B) ratio was higher in the demented patients than the non-demented patients (median, interquartile range; 2.1, 1.3–3.0 *vs*. 1.4, 0.8–2.3, *P* = 0.013). The diversity of the gut microbiome was also assessed using the Shannon index and Simpson tests (Fig. S1). The Shannon index was significantly lower in the non-demented patients than the demented patients (1.89, 1.79–1.95 *vs*. 1.92, 1.88–2.03, *P* = 0.028) and the Simpson test showed a similar trend (0.82, 0.80–0.83 *vs*. 0.83, 0.81–0.85, *P* = 0.086).

Regarding the relationships between MRI findings and the gut microbiome, the F/B ratio in patients with SLI was significantly higher than in those without (2.3, 1.5–3.6 *vs*. 1.5, 0.9–2.3, *P* = 0.028), but there were no significant differences with regard to white matter hypersensitivity (WMH), CMBs and cortical superficial siderosis (CSS) (Tables S2–5). Similarly, both the prevalence of ApoE ε4 and the numbers of patients taking anti-dementia drugs were not associated with the prevalence of either enterotype or the F/B ratio (Tables S6, S7).

### Multivariable analysis

Stepwise multivariable logistic regression analyses (model 1 included enterotype I and model 2 included enterotype III) showed that female sex, enterotype, ApoE ε4, SLI or CMBs, high VSRAD score, and the use of anti-dementia drugs are independently associated with the presence of dementia (Table 2). These statistical models were appropriately fitted as multivariable logistic regression analyses (sensitivity, specificity, and area under the receiver operating characteristic curve [AUC]; model 1; 85%, 85%, 0.93; model 2; 88%, 86%, 0.93; respectively). However, when the analyses were repeated without including enterotype as a variable, the AUCs were lower (model 1; 85%, 79%, 0.89; model 2; 79%, 82%, 0.86; respectively). Although the sample size was too small to ensure complete reliability of the logistic regression analysis, a lower prevalence of *Bacteroides* and a higher prevalence of ‘other’ bacteria were associated with higher odds ratios than the traditional dementia biomarkers ApoE ε4, SLI and high VSRAD score.

**Table 2:** Multivariable logistic regression analysis for the presence of dementia.

(*n* = 128)	OR	95% CI	*P*
**Model 1**			
Female sex	17.0	3.8–123.2	<0.001
*ApoE*	3.9	1.1–14.8	0.035
Enterotype I	0.1	0.02–0.33	<0.001
SLI	15.0	2.2–148.7	0.005
CMBs	2.8	0.62–13.8	0.178
VSRAD	3.5	1.8–8.0	<0.001
Anti-dementia drug	4.8	0.9–28.0	0.064
**Model 2**			
Female sex	19.1	3.4–173.3	<0.001
*ApoE*	4.4	1.2–18.3	0.026
Enterotype III	18.5	4.1–121.9	<0.001
CMBs	6.1	1.4–31.7	0.018
VSRAD	4.2	2.0–10.4	<0.001
Alcohol consumption	1.2	0.3–5.1	0.850
Anti-dementia drug	4.6	0.8–31.1	0.086
Anti-hyperglycaemic drug	7.7	1.5–43.6	0.013

Abbreviations: OR, odds ratio; CI, confidence interval.

The prevalence of the dementia was the dependent variable.

Model 1: enterotype I was included.

Model 2: enterotype III was included.

## Discussion

There has been a recent focus on adverse composition of the gut microbiome as a novel risk factor for dementia^6–8^. In the present study, multivariable analyses adjusted for traditional risk factors revealed that a lower prevalence of *Bacteroides* and a higher prevalence of other bacteria are independently and strongly associated with dementia, and these associations are stronger than those for traditional dementia biomarkers. Similar associations have been found in previous studies of patients with carotid stenosis^13^ and coronary artery disease^5^. *Bacteroides* can regulate endothelial cell function and reduce inflammation^7^, which is consistent with our finding of an inverse relationship between the population of this genus and the presence of dementia. Conversely, Vogt *et al*. concluded that a larger population of *Bacteroidetes* and a smaller population of *Bifidobacterium* in the gut of patients with Alzheimer’s disease^8^ is suggestive of a counter-regulatory effect of *Bacteroidetes* and/or a repressive effect of *Bifidobacterium*. This discrepancy may be due to various differences between the studies, such as ethnicity, dietary composition and the criteria used to diagnose dementia. In the earlier study, a CDR of 0.5 was used as the criterion for the diagnosis of early dementia^8^, but we categorised this score as representative of mild cognitive impairment, implying that the patient is not demented but has a high risk of dementia. Thus, it is probably too early to draw a conclusion. Nevertheless, the relationships between some types of gut microbe and systemic arteriosclerotic diseases suggest common underlying mechanisms in the effects of gut microbial composition on multi-organ arteriosclerosis. The diversity of the gut microbiome is an interesting potential mediator, but we cannot evaluate its importance in detail because our methods were not capable of identifying the ‘other’ bacteria.

We speculate that there is a common microvascular arteriosclerotic and/or inflammatory mechanism underlying cardiovascular and cerebrovascular diseases, and dementia, and some changes in the gut microbiome could accelerate this mechanism. Microvascular arteriosclerosis and inflammation are known risk factors for such diseases^4,6,10,11,13,14^. The significant association between SLI and F/B ratio in the present study is consistent with such a mechanism. Previous studies have revealed that a metabolite released by gut microbiota, trimethylamine N oxide (TMAO), is associated with arteriosclerosis and cardiovascular disease^15,16^, directly increases platelet hyperactivity and potentially thrombosis^17^, and may be a modifiable environmental promoter of Alzheimer’s disease^18^. Because metabolites of this type promote arteriosclerosis, inflammation and the immunological response, further assessment of the metabolites released by gut microbes is warranted to elucidate such associations. The reduction in the release of such metabolites, secondary to normalisation of the gut microbiome, may help to prevent these life-threatening diseases.

This study demonstrates a clear relationship between the gut microbiome and dementia in Japanese patients. Although differences in the gut microbial composition is suggested by a smaller population of *Bacteroides* and a larger population of ‘other’ bacteria, further analysis of differences in the composition of the gut microbiome will be important to clarify the gut-brain connection. Our results are consistent with the hypothesis that gut microbes are involved in the development of dementia. Furthermore, multivariable analyses revealed that the sensitivity, specificity, and AUC for models 1 and 2 were sufficient for the purposes of identifying dementia. Furthermore, addition of enterotype to these analyses improved the scores, showing that enterotype is specifically associated with the presence of dementia. Typical data obtained using next-generation sequencing technology for a microbiome study are presented as OTU counts that are complex; only having positive values, being widely dispersed, and having a large number of zeros^19^. In the present study we did not use this approach, but instead categorised the microbiome into three enterotypes. Although none of the patients demonstrated enterotype II in the demented group, we do not regard zero-inflation as a serious potential problem for our study.

The strengths of the present study were the large sample size compared with previous studies^8,12^, and the nutritional and comprehensive cognitive assessments conducted, including the diagnosing of dementia using detailed neuropsychological tests, brain MRI and single photon emission-computed tomography (SPECT) images. We have shown that bacterial enterotypes are independent risk factors for dementia and are associated with higher odds ratios than traditional risk factors.

However, our study also has several limitations. A causal relationship between differences in the gut microbiome and dementia could not be established because of the cross-sectional study design. A study with a relatively small number of patients may be at risk of being statistically under-powered, and indeed the absence of enterotype II among the demented patients may have affected the statistical interpretation. There is also the possibility of selection bias, because this study was performed in a single hospital-based cohort. High-throughput DNA sequencing technology would be useful to identify the specific genera or species of microbes collectively categorised as ‘other’ bacteria by the T-RFLP method. Other factors, such as the release of inflammatory biomarkers^4^, metabolites such as TMAO^18^, and nutritional and dietary parameters^20^, which may have acted as confounding factors, were not assessed. Because the MNA-SF score was lower in demented patients than non-demented patients, we will analyse the diet of the participants in more detail in a forthcoming further study of the gut microbiome. Analysis of the amyloid β precursor protein could also be useful, because a high serum concentration of this factor suggests inflammatory endothelial dysfunction and a risk of cognitive decline^21^. Subtypes of dementia, such as Alzheimer’s disease or frontotemporal lobar degeneration, have not been considered in the present study, because its aim was to determine the relationship between the gut microbiome and dementia; all-cause dementia was defined by simple categorisation on the basis of the MMSE and CDR scores. Likewise, the potential effects of anti-dementia drugs have not been fully considered because of this study design. However, specific types of dementia may have stronger or weaker associations with the composition of the gut microbiome. Further studies that take these factors into consideration are needed to complement our findings.

## Conclusions

We have shown that components of the gut microbiome, in particular *Bacteroides* and ‘other’ bacteria, are independently associated with dementia, and these associations are stronger than those of traditional dementia biomarkers.

## Methods

### Study design

This was a single-center observational study designed to investigate the association between the composition of the gut microbiome and the clinical condition of the patient, assessed using ADL and cognitive function, named Gerontological investigation of microbiome: a longitudinal estimation study (Gimlet study). This study complied with the Declaration of Helsinki and was approved by the Institutional Review Board at the National Center for Geriatrics and Gerontology (NCGG). Informed consent was obtained from all patients and their families before participating in this study. The study is registered with the UMIN Clinical Trials Registry (UMIN000031851).

### Subjects

Between March 2016 and March 2017, we enrolled consecutive patients visiting the memory clinic at the NCGG who agreed to undergo medical assessment of their cognitive function and faecal examination to survey the gut microbiome. Patients were eligible for the study if they: (1) were able to undergo brain MRI; (2) provided informed consent in writing; (3) provided informed consent for the NCGG Biobank to store their clinical data, blood and faecal samples; and (4) were accompanied by a study partner who could assess the condition of the patient. We excluded patients if they: (1) were unable to undergo MRI examination, or the MRI scan could not be evaluated because of movement; (2) had local lesions, such as cerebral infarction, detected by MRI before enrolment, which could significantly affect cognitive functioning; (3) had a history of a major psychological disorder, or current serious or unstable alcohol or drug abuse; (4) had ≤6 years of education; (5) had a history of cancer of the digestive tract; or (6) were judged by an investigator to be ineligible to participate as a study subject (because of the presence of a brain tumour, encephalitis/meningitis, normal pressure hydrocephalus, Huntington’s disease, progressive supranuclear palsy, corticobasal degeneration, multiple system atrophy, subdural hematoma, multiple sclerosis or lower cognitive function due to head injury).

### Baseline assessment

We assessed the following clinical parameters: (1) demographic characteristics, such as age, sex and years of education; (2) the presence of risk factors, such as hypertension, dyslipidaemia, diabetes mellitus, ischaemic heart disease, chronic kidney disease, smoking habits, history of stroke and alcohol consumption; (3) global cognitive function, using MMSE^22^ and CDR^23^; (4) neuropsychology, using the Alzheimer’s Disease Assessment Scale-cognitive subscale^24^, Raven’s Coloured Progressive Matrices^25^; Frontal Assessment Battery^26^ and Logical Memory subtests I and II of the Wechsler Memory Scale-Revised^27^; (5) laboratory variables, including ApoE ε4 as a risk factor for AD; (6) ankle brachial index and pulse wave velocity as indicators of arteriosclerosis^28^, and the ‘impact’ of pulse^14^; and (7) brain imaging (MRI and SPECT). The clinical samples and data were provided by the NCGG Biobank, which collects clinical data for research. Detailed information is provided in the Supplementary File.

### Comprehensive geriatric assessment

All participants underwent a comprehensive geriatric assessment using: (1) the Barthel Index^29^ to assess basic ADL, and the Lawton and Brody scale to assess instrumental ADL^30^; (2) MMSE and CDR to assess global cognitive function; (3) the Geriatric Depression Scale^31^ to exclude depressive status; (4) the Dementia Behaviour Disturbance Scale^32^ to assess behavioural and psychological symptoms; (5) the Vitality Index to measure vitality in elderly patients; (6) Zarit Caregiver Burden Interview^33^ to assess the burden of caregivers; (7) assessment of movement parameters, such as gait speed, timed up and go test^34^ and fall history; (8) assessment of other items, such as the presence of frailty and hearing loss; (9) assessment of social and lifestyle factors, such as using the MNA-SF to assess nutritional status^35^; and (10) assessment of current medication (anti-dementia drugs, anti-hypertensive drugs, statins, proton pump inhibitors/H2 blockers, anti-thrombotic drugs, anti-hyperglycaemic drugs and aperients).

### Classification of cognitive function

We divided patients into two categories according to their MMSE and CDR scores: (1) a non-demented group (MMSE ≥20 and CDR <1) and a demented group (MMSE <20 and/or CDR ≥1), because these measures reliably indicate the presence of dementia. A CDR score of 0.5 is regarded as indicating the presence of MCI and possibly very mild dementia, meaning that the patient has a higher risk of dementia^36^. Therefore, we categorised a CDR score of 0.5 as representing mild cognitive impairment and included these patients in the non-demented group in the present study.

### Brain imaging

Patients underwent 1.5T MRI of the brain (Philips Ingenia, Eindhoven, the Netherlands). MRI scans, including diffusion-weighted imaging, fluid-attenuated inversion recovery imaging, T2-weighted imaging, T2^*^-weighted gradient echo imaging, 3D T1-weighted sagittal and axial coronal views, and 3D time-of-flight MR angiography scans, were obtained. The presence and components of cerebral small vessel disease were categorised using the standards for reporting vascular changes on neuroimaging^37^, including recent subcortical small infarcts, SLIs, WMH, CMBs and CSS. We used VSRAD advance (Eisai Co., Ltd., Tokyo, Japan) software to quantify cortical and hippocampal atrophy using a standardised z-score.

Patients also underwent N-isopropyl-p-[^123^I]-iodoamphetamine-SPECT. We assessed the presence or absence of a reduction in blood flow in the area of the posterior cingulate gyrus and/or precuneus as a surrogate marker of Alzheimer’s disease^38^.

### Gut microbiome

Faecal samples were collected at home while patients were consuming their usual diet and were frozen and preserved at −81 °C at the NCGG Biobank. After all the samples had been collected, the gut microbiome was analysed using T-RFLP analysis by the TechnoSuruga Laboratory (Shizuoka, Japan)^39^. Microbial DNA was extracted from the faecal samples and amplified by polymerase chain reaction. The resulting 16S rDNA amplicons were treated with *Bsl*I (New England BioLabs, Ipswich, MA, USA). T-RFLP analysis is one of the most well-established and reliable 16S ribosomal RNA-based methods, especially when considering its high throughput and reproducibility (see Supplementary Methods). First, T-RFLP was used to classify gut microbes into the following 10 groups: *Prevotella, Bacteroides*, Lactobacillales*, Bifidobacterium, Clostridium* cluster IV*, Clostridium* subcluster XIVa*, Clostridium* cluster IX*, Clostridium* cluster XI, *Clostridium* cluster XVIII, and ‘others’^5^. Second, we stratified the gut microbiome into three enterotypes: enterotype I included *Bacteroides* at >30%, enterotype II included *Prevotella* at >15% and enterotype III included the remaining bacteria, by reference to the Human Faecal Microbiome T-RFLP profile^5,40^. A recent metagenomic analysis defined these three major clusters of gut microbes in humans on the basis of the predominant bacterial genera present in faecal specimens^5^. This classification was based on the previously established phylogenetic profile similarities obtained by mapping metagenomic reads to 1,511 reference genomes. Multidimensional cluster analysis and principal component analysis showed three distinct clusters, which were designated enterotypes I to III^40^. Enterotype III can also be characterised by the presence of few *Bacteroides* and *Prevotella*, rather than by a dominant genus^5^.

Third, we assessed the F/B ratio^5^. The phylum Firmicutes includes the Lactobacillales and the *Clostridium* clusters, and the phylum Bacteroidetes includes *Bacteroides* and *Prevotella*.

### Statistical analysis

Continuous, ordinal and categorical variables are expressed as mean ± standard deviation, median and interquartile range, and frequency or proportion (percentage), and were compared using the unpaired Student *t*-test, Wilcoxon rank-sum test and χ^2^ test, respectively. First, the composition of the gut microbiome was analysed using a dendrogram and the T-RFLP patterns were analysed using Euclidean distance and the Ward Method. Second, we divided patients into two groups according to the presence or absence of dementia and compared their clinical characteristics using the Wilcoxon signed-rank test and the χ^2^ test. Third, we compared the composition of the gut microbiome between the two groups using the results of the T-RFLP analysis. In particular, the numbers of patients with enterotypes I, II and III were compared between the dementia and non-dementia groups using the χ^2^ tests. We then evaluated the differences in particular taxa using the Wilcoxon signed-rank test. The diversity of the gut microbiome was also assessed using the Shannon and the Simpson tests.

Next, we used multivariable logistic regression models to identify the variables independently associated with dementia. Backward stepwise multivariable logistic regression analyses were performed, adjusting for patient demographics (age, sex and education years), gut microbiota (enterotype I was included in model 1, enterotype III in model 2, and the F/B ratio in both), risk factors (hypertension, diabetes mellitus, dyslipidaemia, chronic kidney disease, ischaemic heart disease, history of stroke, smoking habit, alcohol drinking habit and ApoE ε4), the MNA-SF, brain MRI findings (SLI, WMH, CMBs, CSS and VSRAD score), blood flow reduction on SPECT images (posterior cingulate gyrus and/or precuneus), and current medication (anti-dementia drugs and anti-hyperglycaemic drugs). The sensitivity, specificity and AUC were also calculated to evaluate the usefulness of the technique for identification of the presence of dementia. Odds ratios are presented with 95% confidence intervals. All comparisons were two-tailed, and *P* < 0.05 was considered to represent statistical significance. All data were analysed using the JMP 11.0 software package (SAS Institute Inc., Cary, NC).

## Supplementary information

Supplemental_file

## Data Availability

The datasets used and/or analysed during the current study are available from the corresponding author on reasonable request.
