# Supplementary material for: Analysis of the relationship between the gut microbiome and dementia: a cross-sectional study conducted in Japan
Source: Sci Rep. 2019 Jan 30;9:1008. doi: 10.1038/s41598-018-38218-7 (PMC6353871; doi:10.1038/s41598-018-38218-7)
Supplement: Supplementary file 1 — Supplemental_file [file 41598_2018_38218_MOESM1_ESM.docx]

**Supplementary files**

**Analysis of the relationship between the gut microbiome and dementia: a cross-sectional study conducted in Japan**

Naoki Saji^*1)^, Shumpei Niida^2)^, Kenta Murotani^3)^, Takayoshi Hisada^4)^, Tsuyoshi Tsuduki^5)^, Taiki Sugimoto^1)^, Ai Kimura^1)^, Kenji Toba^1)^, Takashi Sakurai^1,6)^

1. Center for Comprehensive Care and Research on Memory Disorders, National Center for Geriatrics and Gerontology, Aichi, Japan
2. Medical Genome Center, National Center for Geriatrics and Gerontology, Aichi, Japan
3. Biostatistics Center, Graduate School of Medicine, Kurume University, Fukuoka, Japan
4. TechnoSuruga Laboratory Co., Ltd, Shizuoka, Japan
5. Laboratory of Food and Biomolecular Science, Department of Bioscience and Biotechnology for Future Bioindustries, Graduate School of Agricultural Science, Tohoku University, Miyagi, Japan
6. Department of Cognition and Behavioural Science, Nagoya University Graduate School of Medicine, Aichi, Japan

**Supplementary methods**

***MRI***

Patients underwent 1.5T MRI of the brain (Signa EXCITE XL ver. 11.0: GE Healthcare, Milwaukee, WI, USA), including diffusion-weighted imaging, fluid-attenuated inversion recovery (FLAIR) imaging, T2^*^-weighted gradient echo imaging (T2WI) and 3D time-of-flight MR angiography. A recent small subcortical infarct (RSSI) was defined as an infarct of <15 mm in diameter, detected using diffusion-weighted imaging. A silent lacunar infarct was defined as a focal lesion of ≥3 mm in diameter, which was hyperintense on T2WI and hypointense on FLAIR images. White matter hyperintensity was defined as an irregular periventricular hyperintensity (Fazekas grade ≥3) and/or early confluent or confluent separate deep hyperintense lesions (Fazekas grade ≥2) in the white matter on T2WI and FLAIR images. A cerebral microbleed was defined as a focal area of signal loss in the brain parenchyma of <5 mm on a T2^*^ scan. Cortical superficial siderosis was defined as a homogeneous hypointense curvilinear signal intensity (black) on a T2^*^ scan in the superficial layers of the cerebral cortex, within the subarachnoid space, or both.

***Risk factors***

Hypertension was defined by a systolic blood pressure of ≥ 140 mmHg or a diastolic blood pressure of ≥ 90 mmHg and/or the use of anti-hypertensive drugs. Dyslipidaemia was defined by a serum low-density lipoprotein cholesterol concentration of ≥ 140 mg/dL, a serum high-density lipoprotein cholesterol concentration of < 40 mg/dL, a serum triacylglycerol concentration of ≥150 mg/dL, and/or the use of statins. Diabetes mellitus was defined by a haemoglobin A1c (HbA1c) concentration of ≥ 6.5% and/or the use of oral hypoglycaemic drugs or insulin, and/or a fasting serum glucose concentration of ≥ 69.9 mol/L (126 mg/dL). Ischaemic heart disease was defined by the existence of a history of physician-diagnosed *angina pectoris*, and/or evidence of a prior myocardial infarction or a previous coronary revascularisation procedure (percutaneous coronary intervention or coronary artery bypass surgery). Serum creatinine was measured and estimated glomerular filtration rate (eGFR) was determined using the equation proposed by the Japanese Society of Nephrology, as follows: eGFR (mL/min/1.73 m^2^) = 194 × (serum creatinine [mg/dL])^−1.094^ × (age [years])^−0.287^ (× 0.739 if female). CKD was defined by an eGFR of <60 mL/min/1.73 m^2^.

***Sample collection***

Patients or their family members used a faecal sampler to collect a faecal sample as soon as possible after the patient’s bowel movement, which was placed in a specimen container. Patients collected faecal samples on the date of the hospital consultation and presented them to the clinical laboratory centre of the NCGG (preferably within 6 h of bowel movement). After the samples were frozen, they were stored at −81°C at the NCGG Biobank. After all the samples had been collected, they were transported to the TechnoSuruga Laboratory (Shizuoka, Japan), keeping them frozen.

**Terminal restriction fragment length polymorphisms**

Faecal samples (approximately 10 mg each) were suspended in 900 μL of a solution containing 100 mM tris-HCl (pH 9.0), 40 mM ethylenediaminetetraacetic acid, 4 M guanidine thiocyanate and 0.001% bromothymol blue. Faecal solids in the suspension were broken down using a FastPrep FP100A Instrument (MP Biomedicals; CA, USA) and zirconia beads at 5 m/s for 2 min. DNA was then extracted from 200 µL of suspension using an automatic nucleic acid extractor and MagDEA® DNA 200 (Precision System Science, Chiba, Japan). PCR was performed using a Takara Thermal Cycler Dice TP650 (Takara Bio, Shiga, Japan) in a reaction mixture (20 µl) containing 1×PCR buffer, each deoxynucleoside triphosphate at a concentration of 200 µM, 1.5 mM MgCl_2_, each primer at a concentration of 0.2 µM, 10 ng of faecal DNA and 0.2 U of HotStar Taq DNA polymerase (Qiagen, Hilden, Germany). The primers used were 5' fluorescein amidite-labelled 516f (5'-TGC- CAGCAGCCGCGGTA-3'; *Escherichia coli* positions 516–532) and 1510r (5'-GGTTACCTTGTTACGA- CTT-3'; *E. coli* positions 1510–1492). The amplification programme used was as follows: preheating at 95°C for 15 min; 35 cycles of denaturation at 95°C for 30 s, annealing at 50°C for 30 s and extension at 72°C for 90s; and finally, a terminal extension at 72°C for 10 min. The size of the amplicon was verified by electrophoresis and it was purified using a MultiScreen PCR96 Filter Plate (Millipore, Billerica, MA, USA). The purified 16S rDNA amplicons were treated with 10 U of FastDigest *BseLI* (Thermo Fisher Scientific., MA, USA) for 10 min. An ABI PRISM 3130×l genetic analyser (Thermo Fisher Scientific) was used to analyse the resultant fluorescence-labelled terminal restriction fragments (T-RFs), and GeneMapper software (Thermo Fisher Scientific) was used to determine T-RF length and peak area for each sample. T-RFs were divided into 29 operational taxonomic units (OTUs). The OTUs were quantified as the percentage of each OTU of the total OTU area and expressed as the percentage of the area under the curve (% AUC). The reference database, Human Faecal Microbiota T-RFLP profiling (http://www.tecsrg-lab.jp/), was used to putatively identify the bacteria in each classification unit and the corresponding OTU.

**Supplementary results**

| **Table S1. Supplementary patient characteristics** | | | | |
| --- | --- | --- | --- | --- |
|  | Total |  |  |  |
|  |  | Demented | Non-Demented | *P* |
|  | (*n*=128) | (*n*=34) | (*n*=94) |  |
| ***Arterial stiffness*** | | | | |
| Pulse wave velocity | 18.72, 16.0–22.0 | 19.1, 16.4–21.5 | 18.5, 15.9–22.0 | 0.788 |
| Ankle brachial index | 1.10, 1.05–1.15 | 1.1, 1.03–1.14 | 1.11, 1.06–1.15 | 0.385 |
| ***Laboratory findings*** | | | | |
| eGFR, mL/min/1.73 m^2^ | 70.3, 55.7–74.9 | 61.7, 48.2–72.7 | 70.7, 57.7–80.5 | 0.028 |
| ***Medication*** | | | | |
| Anti-dementia drug, n (%) | 15 (11.7) | 9 (25.7) | 6 (6.5) | 0.005 |
| Anti-hyperglycaemic drug, n (%) | 16 (12.6) | 8 (23.5) | 8 (8.6) | 0.035 |
| Anti-hypertensive drug, n (%) | 69 (54.3) | 20 (58.8) | 49 (52.7) | 0.554 |
| Statin, n (%) | 48 (37.8) | 15 (44.1) | 33 (35.5) | 0.412 |
| Anti-thrombotic drug, n (%) | 26 (20.5) | 8 (23.5) | 18 (19.4) | 0.624 |
| PPI/H2 blocker, n (%) | 31 (24.4) | 9 (26.5) | 22 (23.7) | 0.816 |
| Aperient, n (%) | 15 (11.8) | 3 (8.8) | 12 (12.9) | 0.758 |

The Wilcoxon signed-rank and χ2 tests were used.

Anti-dementia drugs: donepezil, rivastigmine, galantamine and memantine.

Anti-hypertensive drugs: calcium channel blockers, angiotensin-converting-enzyme inhibitors and angiotensin II receptor blockers.

PPI: proton pump inhibitor.

| **Table S2. Comparison of characteristics between patients with silent lacunar infarct(s) (SLI) and those without** | | | | | |
| --- | --- | --- | --- | --- | --- |
|  | Total |  |  | |  |
|  |  | SLI (+) | SLI (−) | | *P* |
|  | (*n*=128) | (*n*=14) | (*n*=114) | |  |
| ***Gut microbiome*** | | | | | |
| Enterotype |  |  |  | 0.100 | |
| Enterotype I | 47 (36.7) | 2 (14.3) | 45 (39.5) |  | |
| Enterotype II | 5 (3.9) | 0 | 5 (4.4) |  | |
| Enterotype III | 76 (59.4) | 12 (85.7) | 64 (56.1) |  | |
| F/B ratio | 1.5, 1.0–2.4 | 2.3, 1.5–3.6 | 1.5, 0.9–2.3 | 0.028 | |

The Wilcoxon signed-rank and χ2 tests were used.

| **Table S3. Comparison of characteristics between patients with white matter hyperintensity and those without** | | | | |
| --- | --- | --- | --- | --- |
|  | Total |  |  |  |
|  |  | WMH (+) | WMH (−) | *P* |
|  | (*n*=128) | (*n*=33) | (*n*=95) |  |
| ***Gut microbiome*** | | | | |
| Enterotype |  |  |  | 0.266 |
| Enterotype I | 47 (36.7) | 16 (48.5) | 31 (32.6) |  |
| Enterotype II | 5 (3.9) | 1 (3.0) | 4 (4.2) |  |
| Enterotype III | 76 (59.4) | 16 (48.5) | 60 (63.2) |  |
| F/B ratio | 1.5, 1.0–2.4 | 1.4, 0.7–2.8 | 1.5, 1.0–2.4 | 0.738 |

The Wilcoxon signed-rank and χ2 tests were used.

| **Table S4. Comparison of characteristics between patients with cerebral microbleeds (CMBs) and those without** | | | | |
| --- | --- | --- | --- | --- |
|  | Total |  |  |  |
|  |  | CMBs (+) | CMBs (−) | *P* |
|  | (*n*=128) | (*n*=27) | (*n*=101) |  |
| ***Gut microbiome*** | | | | |
| Enterotype |  |  |  | 0.912 |
| Enterotype I | 47 (36.7) | 9 (33.3) | 38 (37.6) |  |
| Enterotype II | 5 (3.9) | 1 (3.7) | 4 (4.0) |  |
| Enterotype III | 76 (59.4) | 17 (63.0) | 59 (58.4) |  |
| F/B ratio | 1.5, 1.0–2.4 | 2.1, 0.8–3.3 | 1.5, 1.0–2.3 | 0.319 |

The Wilcoxon signed-rank and χ2 tests were used.

| **Table S5. Comparison of characteristics between patients with cortical superficial siderosis (CSS) and those without** | | | | |
| --- | --- | --- | --- | --- |
|  | Total |  |  |  |
|  |  | CSS (+) | CSS (−) | *P* |
|  | (*n*=128) | (*n*=8) | (*n*=120) |  |
| ***Gut microbiome*** | | | | |
| Enterotype |  |  |  | 0.840 |
| Enterotype I | 47 (36.7) | 3 (37.5) | 44 (36.7) |  |
| Enterotype II | 5 (3.9) | 0 (0) | 5 (4.2) |  |
| Enterotype III | 76 (59.4) | 5 (62.5) | 71 (59.2) |  |
| F/B ratio | 1.5, 1.0–2.4 | 1.9, 0.8–2.7 | 1.5, 1.0–2.4 | 0.716 |

The Wilcoxon signed-rank and χ2 tests were used.

| **Table S6. Comparison of characteristics between patients with ApoE ε4 and those without** | | | | |
| --- | --- | --- | --- | --- |
|  | Total |  |  |  |
|  |  | *ApoE* (+) | *ApoE* (−) | *P* |
|  | (*n*=128) | (*n*=39) | (*n*=89) |  |
| ***Gut microbiome*** | | | | |
| Enterotype |  |  |  | 0.724 |
| Enterotype I | 47 (36.7) | 13 (33.3) | 34 (38.2) |  |
| Enterotype II | 5 (3.9) | 1 (2.6) | 4 (4.5) |  |
| Enterotype III | 76 (59.4) | 25 (64.1) | 51 (57.3) |  |
| F/B ratio | 1.5, 1.0–2.4 | 1.5, 0.9–2.3 | 1.6, 1.0–2.7 | 0.406 |

The Wilcoxon signed-rank test and χ2 tests were used.

| **Table S7. Comparison of characteristics between patients with anti-dementia drugs and those without** | | | | |
| --- | --- | --- | --- | --- |
|  | Total |  |  |  |
|  |  | *Anti-dementia drugs* (+) | *Anti-dementia drugs* (-) | *P* |
|  | (*n*=128) | (*n*=15) | (*n*=113) |  |
| ***Gut microbiome*** | | | | |
| Enterotype |  |  |  | 0.700 |
| Enterotype I | 47 (36.7) | 6 (40.0) | 41 (36.3) |  |
| Enterotype II | 5 (3.9) | 0 (0) | 5 (4.4) |  |
| Enterotype III | 76 (59.4) | 9 (60.0) | 67 (59.3) |  |
| F/B ratio | 1.5, 1.0–2.4 | 2.4, 0.7–3.1 | 1.5, 1.0–2.3 | 0.265 |

The Wilcoxon signed-rank test and χ2 tests were used.

**Figure S1.**  **The diversity of the gut microbiome assessed using the Shannon and the Simpson tests**
